# Wearable technologies for assisted mobility in the real world

**DOI:** 10.1038/s41467-025-67126-4

**Published:** 2025-12-08

**Authors:** Shuo Gao, Jianan Chen, Yunjia Xia, Xuemeng Li, Weihao Ma, Huixin Yang, Jinchen Li, Xinkai Zhou, Tianyu Jia, Yuchen Xu, Julie Uchitel, Dean Ta, Peng Qi, Junbo Ge, Yi Guo, Yajie Qin, Inseung Kang, Wenyao Xu, He Li, Jinke Chang, Siming Zuo, Shiwei Wang, Shan Luo, Letizia Gionfrida, Chen Hu, Shuqin Dong, Yongxin Guo, Yixuan Yuan, Haixia Zhang, Haotian Chen, Yu Pan, Chenyun Dai, Qinyuan Ren, Rui Loureiro, Tom Carlson, Wei Chen, Yuanting Zhang, Panicos Kyriacou, Hadi Heidari, Kia Nazarpour, Themis Prodromakis, Alexander Casson, Tamar R. Makin, Gert Cauwenberghs, Dario Farina, Hubin Zhao

**Affiliations:** 1 School of Instrumentation and Optoelectronic Engineering, Beihang University, Beijing, China; 2 HUB of Intelligent Neuro-engineering, CREATe, Division of Surgery and Interventional Science, UCL, London, UK; 3 Department of Bioengineering, Imperial College London, London, UK; 4 Shu Chien-Gene Lay Department of Bioengineering, University of California San Diego, La Jolla, CA USA; 5 School of Medicine, Stanford University, Stanford, CA USA; 6 College of Biomedical Engineering, Fudan University, Shanghai, China; 7 Department of Control Science and Engineering, College of Electronic and Information Engineering, Tongji University, Shanghai, China; 8 Department of Cardiology, Zhongshan Hospital, Fudan University, Shanghai Institute of Cardiovascular Diseases, Shanghai, China; 9 Department of Mechanical Engineering, Carnegie Mellon University, Pittsburgh, PA USA; 10 The Embedded Sensing and Computing (ESC) group, Department of Computer Science & Engineering, University at Buffalo, The State University of New York, Buffalo, NY USA; 11 School of Electronic Science and Engineering, Southeast University, Nanjing, Jiangsu China; 12 Multifunctional Materials and Composites (MMC) Laboratory, Department of Engineering Science, University of Oxford, Oxford, UK; 13 Microelectronics Lab (meLAB), James Watt School of Engineering, University of Glasgow, Glasgow, UK; 14 Centre for Electronics Frontiers, Institute for Integrated Micro and Nano Systems, School of Engineering, University of Edinburgh, Edinburgh, UK; 15 Robot Perception Lab (RPL), Department of Engineering, King’s College London, London, UK; 16 Vision in Human Robotics Lab, Department of Informatics, King’s College London, London, UK; 17 State Key Laboratory of Terahertz and Millimeter Waves, Department of Electrical Engineering, City University of Hong Kong, Hong Kong SAR, China; 18 Department of Electrical and Computer Engineering, National University of Singapore, Singapore, Singapore; 19 Department of Electronic Engineering, The Chinese University of Hong Kong, Hong Kong SAR, China; 20 National Key Laboratory of Science and Technology on Micro/Nano Fabrication, Beijing Advanced Innovation Center for Integrated Circuits, School of Integrated Circuits, Peking University, Beijing, China; 21 HiMEX Lab, James Watt School of Engineering, University of Glasgow, Glasgow, UK; 22 Department of Rehabilitation Medicine, School of Clinical Medicine, Tsinghua University, Beijing, China; 23 School of Biomedical Engineering, Shanghai Jiao Tong University, Shanghai, China; 24 Institute of Industrial Engineering, College of Control Science and Engineering, Zhejiang University, Hangzhou, Zhejiang China; 25 School of Biomedical Engineering, University of Sydney, Sydney, Australia; 26 Research Centre for Biomedical Engineering, School of Science & Technology, City St George’s, University of London, London, UK; 27 Edinburgh Movement and Rehabilitation (MoveR) hub, School of Informatics, University of Edinburgh, Edinburgh, UK; 28 Department of Electrical and Electronic Engineering, University of Manchester, Manchester, UK; 29 MRC Cognition and Brain Sciences Unit, University of Cambridge, Cambridge, UK

**Keywords:** Biomedical engineering, Electrical and electronic engineering

## Abstract

Mobility impairments from aging, injury, or medical conditions limit independence and social participation. Conventional assistive devices lack adaptability in complex environments. Recent wearable technologies integrating neural sensing, electronics, and co-design offer personalized, responsive mobility support. This perspective focuses on advances in wearable sensing and multimodal fusion for intent recognition, environmental interaction, and adaptive control in exoskeletons, prosthetics, smart wheelchairs, and navigation systems. Emphasizing human-in-the-loop and cognitive–sensorimotor integration, it outlines emerging trends and challenges, promoting intelligent, user-centered solutions to restore function and enhance autonomy, accessibility, and inclusion for individuals with mobility impairments.

## Abstract (web summary)

Mobility impairment reduces autonomy and social participation. Here, the authors discuss how wearable technologies are transforming assisted mobility towards more adaptive, intuitive, and user-centered solutions.

## Introduction

Approximately 1.3 billion people live with significant disabilities, about 1 in 6 individuals^1^. Complementing this, data from the United Nations Statistics Division^2^ indicate that ~58 million people worldwide live with some form of walking or mobility impairment. Assistive technologies have emerged to enable individuals to regain some degree of functional independence. While traditional assistive devices such as wheelchairs, crutches, and prostheses have significantly improved mobility, they often lack the flexibility, adaptability, and comfort required for effective use in dynamic, real-world environments^3–7^.

Recent advancements, particularly in wearable technologies, are driving a transformative shift in the field of assistive mobility. Modern wearable devices offer an enhanced user experience through their emphasis on adaptability, comfort, and user-centered design. Wearables can dynamically adjust to the user’s needs in real-time, offering unprecedented levels of mobility support previously unattainable with conventional approaches^8–10^.

**Fig. 1:**
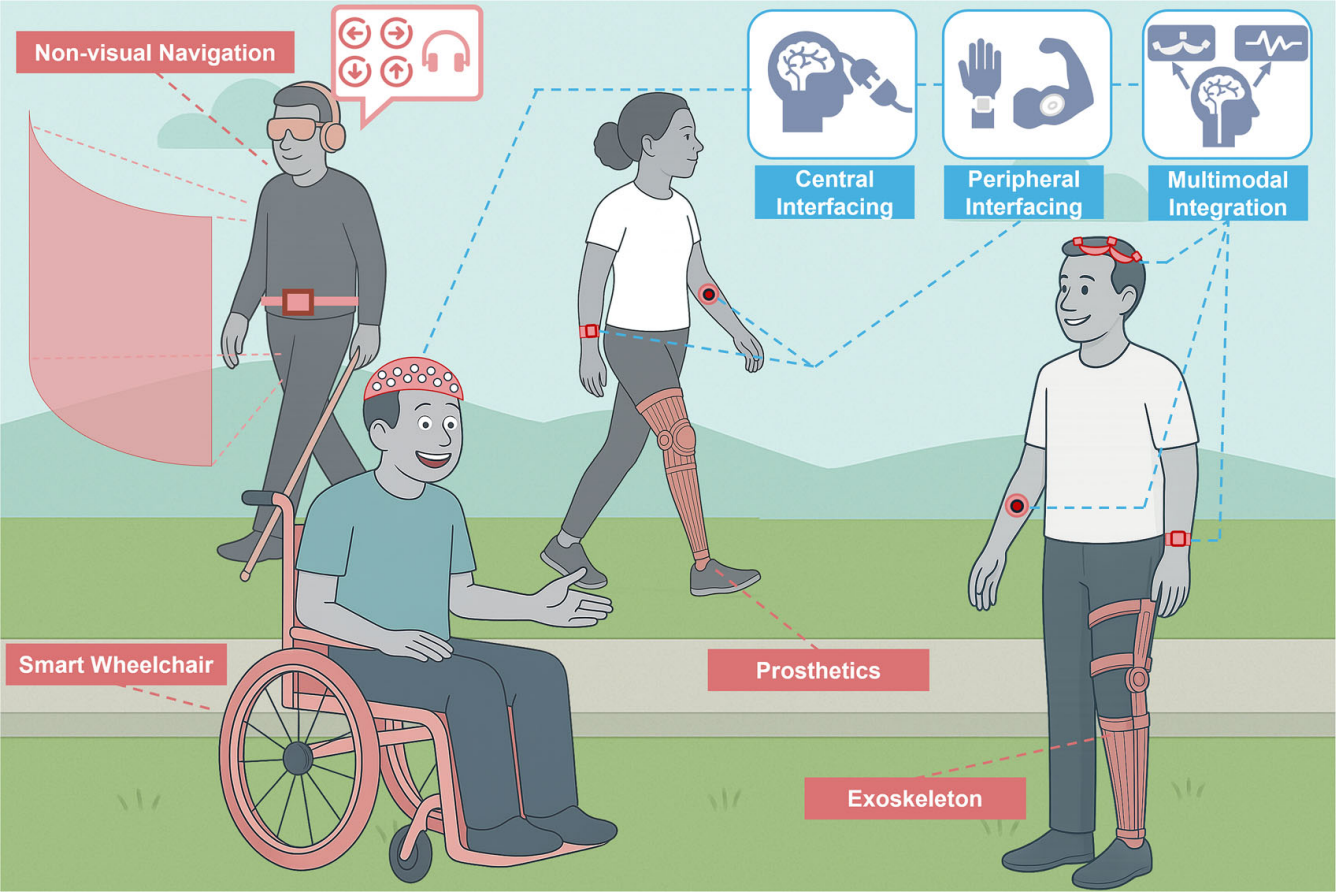
Real-world applications of wearable technologies for assisted mobility. The figure illustrates primary applications of assisted mobility, i.e., exoskeletons, prosthetics, smart wheelchairs, and non-visual navigation aids, using wearable sensing technologies. These integrated solutions support diverse user needs in mobility and navigation across outdoor environments.

The field of wearable technologies for mobility assistance brings together diverse disciplines, including robotics, computer science, neuroscience, and brain-computer interfaces (BCIs)/human-robot interaction. As illustrated in Fig. 1, this interdisciplinary convergence has enabled the seamless integration of wearable sensing modalities—such as electroencephalography (EEG), functional near-infrared spectroscopy (fNIRS), electromyography (EMG), electrooculography (EOG), and inertial motion sensors—into assistive devices. This integration has paved the way for adaptive, user-responsive systems that can dynamically interpret and respond to the user’s physiological and behavioral signals. As these technologies advance, the incorporation of co-creation design, multimodal integration, and human-in-the-loop strategies is poised to drive more user-centered development and enhance the functionality and reliability of assistive devices. Together, these innovations mark a paradigm shift, enabling more effective, intuitive mobility solutions that integrate seamlessly into everyday life.

**Fig. 2:**
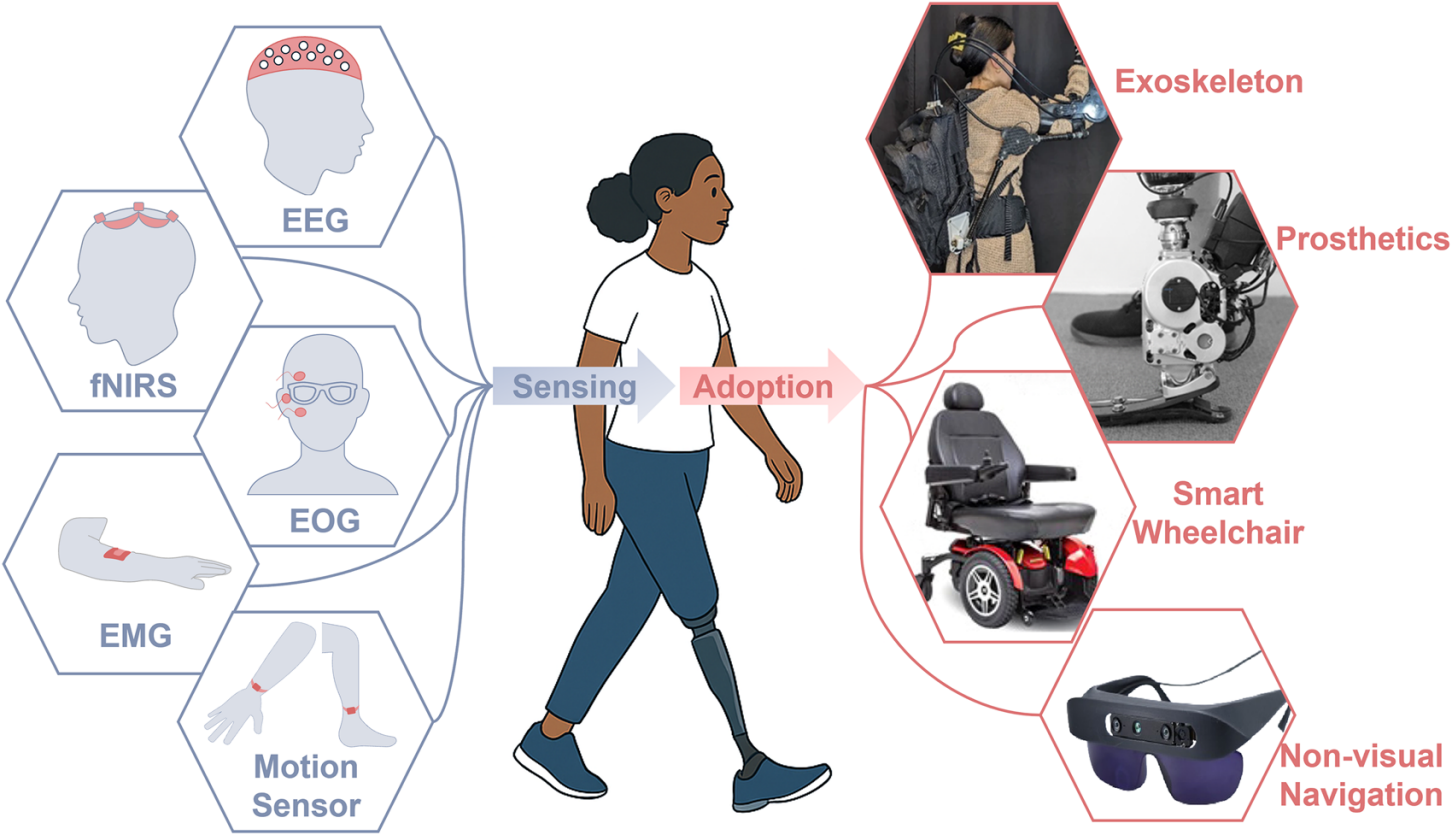
Wearable sensing technologies and their enabling roles in assisted mobility applications. This diagram highlights key modalities—EEG, fNIRS, EOG, EMG, and motion sensing—and their multimodal integration in supporting real-world applications such as exoskeletons, prosthetics, smart wheelchairs, and non-visual navigation. Sensor placement across the body reflects diverse interaction points tailored to user intent recognition and environmental awareness. Reused with permission from Tang et al.^166^.

This perspective provides a focused overview on the role of wearable technologies—encompassing neural, physiological, and kinematic sensing—in assisted mobility, excluding other categories of assistive devices such as stationary rehabilitation systems or sensors that perceive and respond to environmental conditions (as shown in Fig. 2). Unlike previous reviews^11–14^ that broadly survey wearable systems, we emphasize the unique challenges, current progress, and future potential of wearable solutions designed to enhance mobility and independence for individuals with motor impairments. This perspective also presents an argument as to why the integration of diverse sensing technologies is essential for the continued development of effective assistive mobility technologies.

The scope is deliberately focused on lightweight, user-adaptive devices designed for real-time interaction with the human body in real-world environments. These include, but are not limited to, exoskeletons, prosthetics, smart wheelchairs, and non-visual navigation systems. Furthermore, each technology discussed is either already commercialized or shows a well-defined and realistic pathway toward scalable production and market entry. This perspective aims to serve as a practical guide for researchers, engineers, and clinicians engaged in the development and deployment of assistive mobility technologies.

## Current landscape of wearable human sensing technologies

### Decoding user intent

Effective assistive mobility requires a direct interface with the human nervous system—typically through estimating user intent—to enable intuitive and responsive control. Accurate recognition and interpretation of user intent are essential for intuitive and responsive control in assistive mobility devices, ensuring the system aligns with the user’s movement intentions. Intent recognition can be broadly classified into central and peripheral interfacing^15^. Central interfacing methods interpret cognitive intent by capturing brain activity through signals such as EEG or fNIRS. Peripheral interfacing relies on physiological and motion-based signals, including EMG, eye tracking, EOG, and inertial measurement units (IMUs), to infer user intent based on neuromuscular and kinematic cues. A more detailed explanation of these sensing modalities can be found in a summary table provided in Table 1^15,16–24^. Rather than directly contributing to intent understanding, these technologies can be utilised to form a complete closed-loop system where effective sensing and mossnitoring is vital. Physiological signals such as electrocardiogram (ECG), photoplethysmography (PPG), heart rate variability, blood pressure, electrodermal activity, breathing, sweat biomarkers are then generally reactive physiological indicators; they reflect physiological changes resulting from, rather than preceding, the intended motion. They thus do not necessarily contribute to intent understanding, but may provide additional context and form part of a complete closed-loop system where monitoring success is important. The choice of sensing approach depends on the user needs. Some applications require brain activity monitoring, while others rely on body signals. Integrating central and peripheral interfaces in wearable systems enhances both accuracy and adaptability; this section will outline the key advantages of each modality as well as of their integration.

**Table 1:** Comprehensive Comparison of Sensing Modalities in Neurophysiological and Motion Monitoring

Modality	Signal Source	Core Features (Quantitative)	Limitations (Quantitative/Qualitative)	Engineering Parameters (Sampling Rate / Bandwidth / SNR / Placement / Power, etc.)	Common Evaluation Metrics	Wearability Parameters	Typical Applications
EEG	Brain electrical activity	High temporal resolution; sampling rate ≥256 Hz (commonly 512–2000 Hz); bandwidth 0.5–80 Hz	Sensitive to noise; low spatial resolution; relatively poor SNR	Resolution ≥16 bits (commonly 24 bits); electrode placement based on 10–20 system; power/weight varies by device	Signal-to-noise ratio (SNR), classification accuracy, P300/N200 detection rate	Dry/wet electrodes; cap comfort ( < 200 g); continuous use duration	Brain–computer interface; mental fatigue monitoring; elderly driving safety
fNIRS	Cortical haemodynamic response	High spatial resolution; sampling rate ~8 Hz; bandwidth 0.01–0.2 Hz	Slow response (seconds); sensitive to motion artefacts	Source–detector spacing ≈3 cm; fiber-optic probes; power/weight varies by device	Amplitude of oxygenation change, latency, task classification accuracy	Fiber/LED module weight ( < 200 g); head comfort; battery life (several hours)	MI-based prosthesis control; cognitive workload monitoring; elderly brain health training
EMG	Muscle electrical activity	Bandwidth 10–500 Hz; amplitude 0.1–2000 mV	Affected by electrode-skin impedance and muscle fatigue	Noise usually < 10–20 µV; CMRR > 10^6^; weight/power varies by device	Signal amplitude stability, activation latency, movement classification accuracy	Surface electrode adhesion; sweat resistance; module weight ~10–50 g	Exoskeleton control; active prosthesis control; smart rollator integration
EOG	Ocular electrical activity	Low computational load; bandwidth 0.1–100 Hz; sampling rate ≥200 Hz	Low spatial resolution; false trigger rate	Electrodes placed around the eyes; low power ( < 100 mW); lightweight	Gaze detection accuracy, false trigger rate, response latency	Electrode comfort; low irritation; long-term stability	Gaze-based wheelchair/arm control; virtual interaction
IMU	Body motion/acceleration	Portable, non-contact; pronounced drift issues	Accumulated error over time; drift in position estimation	Sampling rate 100–1000 Hz; gyroscope drift rate ≤0.1°/h (high-end); noise density specified; power varies by chip	Drift rate, gait recognition accuracy, fall-detection sensitivity	Module weight 1–10 g; battery life 8–24 h	Gait tracking; balance control; fall detection; context-aware navigation

#### Central interfacing: functional brain imaging

Over the last decade, wearable assistive devices for mobility and performance have increasingly aimed to decode user intent from brain signals^25^, directly leveraging neural activity for device control. This approach aligns with motor control processes, supported by various non-invasive neuroimaging modalities based on distinct physiological principles. EEG records electrical activity using scalp electrodes to detect voltage changes from neuronal ionic currents^26^. Magnetoencephalography, conversely, measures the magnetic fields produced by these currents, offering comparable temporal resolution and improved cortical localization^27^. Functional magnetic resonance imaging (fMRI) tracks localized changes in cerebral blood flow as indicators of neural activation during tasks^28^. Lastly, fNIRS, a potential “wearable alternative” to fMRI, quantifies changes in oxygenated and deoxygenated haemoglobin to monitor cortical activity, albeit with fine spatial resolution. While magnetoencephalography and fMRI provide high spatial resolution for brain activity mapping, their reliance on expensive, stationary equipment^28^ and controlled environments^29^ limits their feasibility for integration with assistive mobility devices such as exoskeletons or smart wheelchairs. In contrast, EEG and fNIRS offer the advantages of being lightweight, cost-effective, and easily constructed into wearable form factors, making them more suitable for real-time user intent recognition in assistive mobility systems.

EEG enables users to control assistive devices via BCIs, bypassing the need for physical input. Two widely used paradigms in EEG-based BCIs are motor imagery (MI) and steady-state visual evoked potentials (SSVEP). MI can vary by movement type, laterality, or motor state (e.g., walking vs. standing), offering precise control ^30^. However, MI requires sustained concentration, and prolonged use may induce mental fatigue^31^. Consequently, monitoring cognitive state is essential for maintaining effective control. This can also be monitored during MI tasks using EEG, with secondary tasks such as mental arithmetic and adaptive feedback employed to sustain attention and assess user engagement^32^. SSVEP-based BCIs use visual stimuli to elicit frequency-specific neural responses. This approach offers precise and rapid command selection with high reliability, minimal user training, and fast response time. For instance, a recent study introduced an augmented reality (AR)-based BCI system using SSVEPs to enable hands-free prosthetic control with eight distinct hand movement modes^33^. However, their reliance on continuous visual input can cause visual fatigue and limit usability in prolonged tasks like wheelchair navigation, where eye strain and reduced situational awareness may compromise safety^34^. Therefore, the selection of control modalities should be tailored to the specific requirements of each application.

fNIRS effectively detects movement-related cortical activation, characterized by increases in oxygenated haemoglobin in the motor cortex during movement preparation and execution. For example, in individuals with transhumeral amputations, one study combined fNIRS with an artificial neural network to classify six upper-limb motion intentions, including elbow extension/flexion, wrist pronation/supination, and hand opening/closing^35^. fNIRS can also monitor MI and cognitive functions such as attention and decision-making, especially in neurorehabilitation settings like gait training^36^. While its high spatial specificity makes it well-suited for tracking cognitive workload, its limited temporal resolution—due to delayed hemodynamic responses—limits its effectiveness for capturing rapid changes.

As previously noted, EEG provides high temporal resolution for real-time detection of movement intent, whereas fNIRS offers superior spatial resolution for localising brain activation. Their complementary strengths make EEG–fNIRS integration a powerful approach to enhance both the accuracy and responsiveness of user intent recognition. Several initial studies combining EEG and fNIRS have explored lower limb motor imagery^37^ and contributed to an improved understanding of gait and balance^38^. Diffuse Optical Tomography (DOT), an advanced offshoot of fNIRS that reconstructs 3D hemodynamic responses, further enhances spatial resolution^39^.

#### Peripheral interfacing

Indirect methods for inferring user movement intent involve monitoring physiological and physical cues, providing useful information into navigational intentions and motor control. Wearable EMG sensors are widely employed in prosthetics and exoskeletons to detect muscle activity^40^, allowing for intuitive and responsive device control. Eye-tracking techniques and EOG-based systems help determine a user’s intended direction^41^. Beyond movement-related signals, physiological indicators such as heart rate, heart rate variability, respiration rate, and galvanic skin response can be integrated to assess the user’s physical state and movement readiness^42^. By capturing real-time physiological and biomechanical data, these indirect methods enhance the adaptability and responsiveness of mobility-assistive wearable devices, enabling more seamless interaction between the user and the assistive device.

**Fig. 3:**
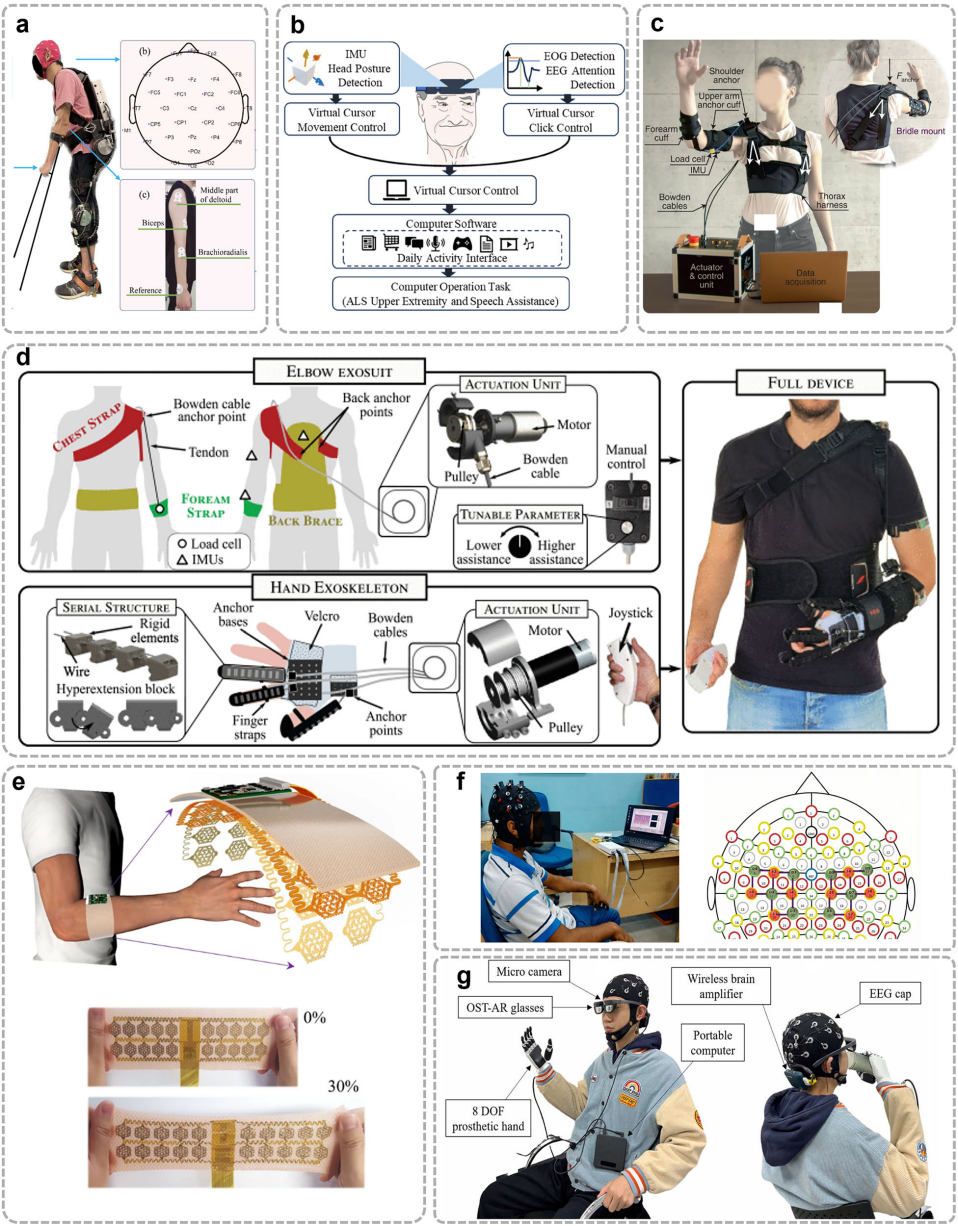
Illustration of wearable sensing technologies. **a** Overview of a lower limb exoskeleton using TFDP, a time-frequency method based on differential pattern analysis for feature extraction^64^. Reused with permission from Li et al.^64^. **b** Wearable BCI mouse system with headband, electrode placement, and schematic control framework^66^. **c** Textile-based exomuscle (Myoshirt) for shoulder support using tendon-driven actuation anchored on the thorax and upper arm^62^. Reused with permission from Geogarakis et al.^62^. **d** Soft upper-limb wearable robots including elbow and hand exosuits with remote actuation and IMU-based torque control^43^. **e** Stretchable high-density EMG sensor enabling real-time gesture recognition *via* AI-based processing^49^. **f** fNIRS-based system for recognizing upper-limb motion intention *via* optodes placed on the motor cortex^35^. **g** Brain-controlled prosthetic hand platform integrating EEG, AR glasses, and an 8-degree of freedom (DOF) prosthesis for real-time control^33^.

EMG captures the electrical signals generated by motor units during skeletal muscle contraction^40^, providing a reliable means to infer user movement intent. By capturing muscle activation patterns, EMG enables real-time decoding of voluntary movements, facilitating intuitive control of assistive devices such as wearable robotic system^43^, wheelchairs^44^ and exoskeletons^45^, as illustrated in Fig. 3. The intensity and frequency of the recorded signals correspond to the degree of muscle activation, enabling the inference of the user’s intention to execute specific movements. Driven by advancements in sensor miniaturization, signal processing algorithms, and material science, EMG systems have evolved to offer greater precision, adaptability, and user comfort^46^. Notably, innovations such as high-density EMG (HD-EMG)^47,48^ (Fig. 3e) and stretchable patches^49,50^ have significantly enhanced the reliability and practicality of electrophysiological sensing in wearable applications.

Eye-tracking and EOG are complementary techniques for monitoring ocular movements. Eye-tracking systems employ infrared cameras to detect pupil position and gaze direction, allowing for precise tracking of a user’s visual attention^51^. Gaze-based control is especially effective in assistive devices like wheelchairs; for instance, a gaze-enabled smart wheelchair aimed at individuals with severe physical impairments such as Amyotrophic Lateral Sclerosis and quadriplegia was demonstrated^52^. Eye-tracking has also proven useful in upper-limb prosthetics, with one study showing that gaze-based wrist movement prediction reduced compensatory shoulder and trunk motions^53^. Commercially available systems such as Tobii Dynavox have already been used^52,54^. In contrast, EOG measures the electrical potential differences generated by ocular movement, utilizing electrodes placed around the eyes to capture these bioelectrical signals^55^. Although EOG-based systems have lower spatial resolution than optical eye-tracking methods, they offer reliable eye movement detection with low computational demand, strong resistance to lighting variations, and easy integration into compact wearable devices^56,57^. EOG is also often used alongside EEG for neural control applications and has been applied to robotic arms^58^ and exoskeletons^59^ to assist stroke survivors with chronic paralysis in performing activities of daily living^58^.

IMUs measure motion-related parameters, including acceleration, angular velocity, and sometimes magnetic orientation, making them essential for wearable gait analysis and body motion tracking^60^. They provide continuous kinematic data for precise assessment of joint angles, stride length, gait phases, and balance. IMUs can measure abnormal gait patterns, enabling adaptive control in exoskeletons. IMUs have also been used in prosthetic knees to assess performance in tasks such as treadmill walking, incline/decline and stair navigation, and obstacle crossing. They allow for the evaluation of gait symmetry, comfort, and functional outcomes in individuals with limb amputations^61^. IMUs can additionally be integrated with advanced soft wearable systems to provide real-time posture monitoring, balance assessment, and adaptive movement assistance, enhancing mobility for individuals with motor impairments^62^.

#### Multimodality

Single-modal sensing methods, such as EEG, EMG, and IMUs, are widely used for motion intent recognition, yet each has inherent limitations. EEG, particularly in motor-impaired users, enables intent decoding via motor imagery but is susceptible to motion artifacts and environmental noise^39^, making it less suitable for dynamic tasks like walking. EMG captures neuromuscular activity preceding movement and is effective for proactive control of exoskeletons. However, its reliability can diminish over time due to muscle fatigue, electrode displacement, and skin impedance variability^63^. IMUs provide valuable kinematic data for estimating posture and joint angles, supporting adaptive locomotion. However, they are prone to drift accumulation over prolonged use, degrading accuracy and increasing processing latency to certain degree during sensor fusion^16^.

The features of individual sensing modalities are often complementary—where one underperforms, another can compensate. Multimodal signal fusion leverages this complementarity by integrating diverse sensor inputs to improve accuracy, responsiveness, and robustness in mobility assistance. Numerous studies have demonstrated that combining brain signals (e.g., EEG, fNIRS) with physiological or motion-based signals (e.g., EMG, EOG, IMUs) enhances intent recognition and system reliability. For example, EMG’s rapid response offsets EEG’s latency, while EEG contributes intent-related information when EMG degrades under fatigue. EEG–EMG integration^64,65^ improves motor intent detection by combining EEG’s early representation of motor planning with EMG’s detailed muscle activation signals, enabling more accurate reconstruction of complex limb movements. Similarly, EEG-EOG fusion^42,59^ supports eye-gaze-based control using EOG for precise eye movement detection and ocular artifact removal, improving EEG signal quality and command accuracy. IMUs further enhance context-awareness and support differentiation between voluntary movements and external disturbances. When combined with EEG^66^ or EMG^43^, IMUs improve motion tracking and control precision in devices such as prosthetics and exoskeletons, particularly in dynamic environments. As noted in Section 2.1.1, EEG-fNIRS fusion also improves classification accuracy and enables cognitive state monitoring, supporting adaptive assistance during rehabilitation^67^.

Effective sensor fusion techniques are essential to fully exploit the complementary strengths of multimodal signals such as EEG, EMG, EOG, and IMUs. Traditional fusion approaches fall into three categories: data-level^37,42^, feature-level^64,65^, and decision-level^65^. Data-level fusion combines raw signals from multiple modalities, preserving maximum information but often encountering issues like noise amplification and signal misalignment. Feature-level fusion extracts modality-specific features and concatenates them for joint modelling, balancing data richness and complexity but requiring precise feature engineering and synchronization. Decision-level fusion integrates outputs from independent classifiers, offering modularity but limiting cross-modal interaction. While these methods can perform well in controlled settings, they often lack joint optimization across modalities and require improvements in accuracy, sensitivity, and generalizability to meet real-world demands^39^.

### Understanding environmental context

Beyond intent detection, environmental perception is essential for assistive mobility devices to adapt to varied terrains, avoid obstacles, and ensure user safety. This capability enables systems like smart wheelchairs, prosthetics and exoskeletons to navigate complex environments while enhancing safety and energy efficiency. A critical aspect of environmental understanding in assistive systems is accurately recognizing user intent within dynamic, task-oriented contexts. This involves not only interpreting physiological signals but also situational cues that reflect the user’s interaction goals. Complementing these approaches, image-based sensing has also proven essential in prosthetic calibration. For example, an image-based calibration system utilizing LED markers and camera-based image analysis enables accurate measurement of joint angles^68^. This ensures anatomically realistic motion calibration during development, which is critical for enabling precise and coordinated hand function. By supporting individualized and visually validated calibration, such systems contribute directly to improving the dexterity, control accuracy, and task-specific adaptability of upper-limb prostheses.

Another key aspect of environmental understanding is obstacle detection and avoidance, which allows mobility devices to identify potential hazards, such as uneven surfaces, staircases, curbs, or moving objects^69^. Alternatively, objects should not always be considered obstacles when planning navigation; for example, the user of a mobility device may wish to approach a person or dock to a table to have an interaction^70^. Technologies such as computer vision (CV)^71,72^ and Red, Green, Blue-Depth cameras^73,74^ provide real-time spatial awareness, enabling precise path planning and responsive adjustments to avoid collisions. This is particularly crucial for smart wheelchairs, lower-limb exoskeletons^74^, and prosthetics^73^, where safe navigation in crowded or dynamic environments is a primary concern^75^. For example, one study demonstrated that CV enhances mobility by enabling a robotic companion to track user movement without requiring body-mounted sensors^72^. Using a 3D vision system to accurately determine the relative position and orientation between the human and the robot, the system provides hands-free, real-time navigation assistance, reducing user effort and promoting independent mobility. Multi-sensor systems combining ultrasonic, passive infrared motion sensors, IMUs, and smartphone-based feedback have also been used to detect obstacles, surface changes, and moving objects, supporting real-time navigation and enhancing mobility for visually impaired users^76^.

## Real-world adoption of wearable technologies for assisted mobility

### Exoskeletons for mobility assistance in rehabilitation medicine

Exoskeletons are advanced wearable robotic devices designed to assist individuals with neurological and musculoskeletal disorders, such as stroke rehabilitation, spinal cord injury (SCI), multiple sclerosis, and Duchenne muscular dystrophy^77–83^.

**Fig. 4:**
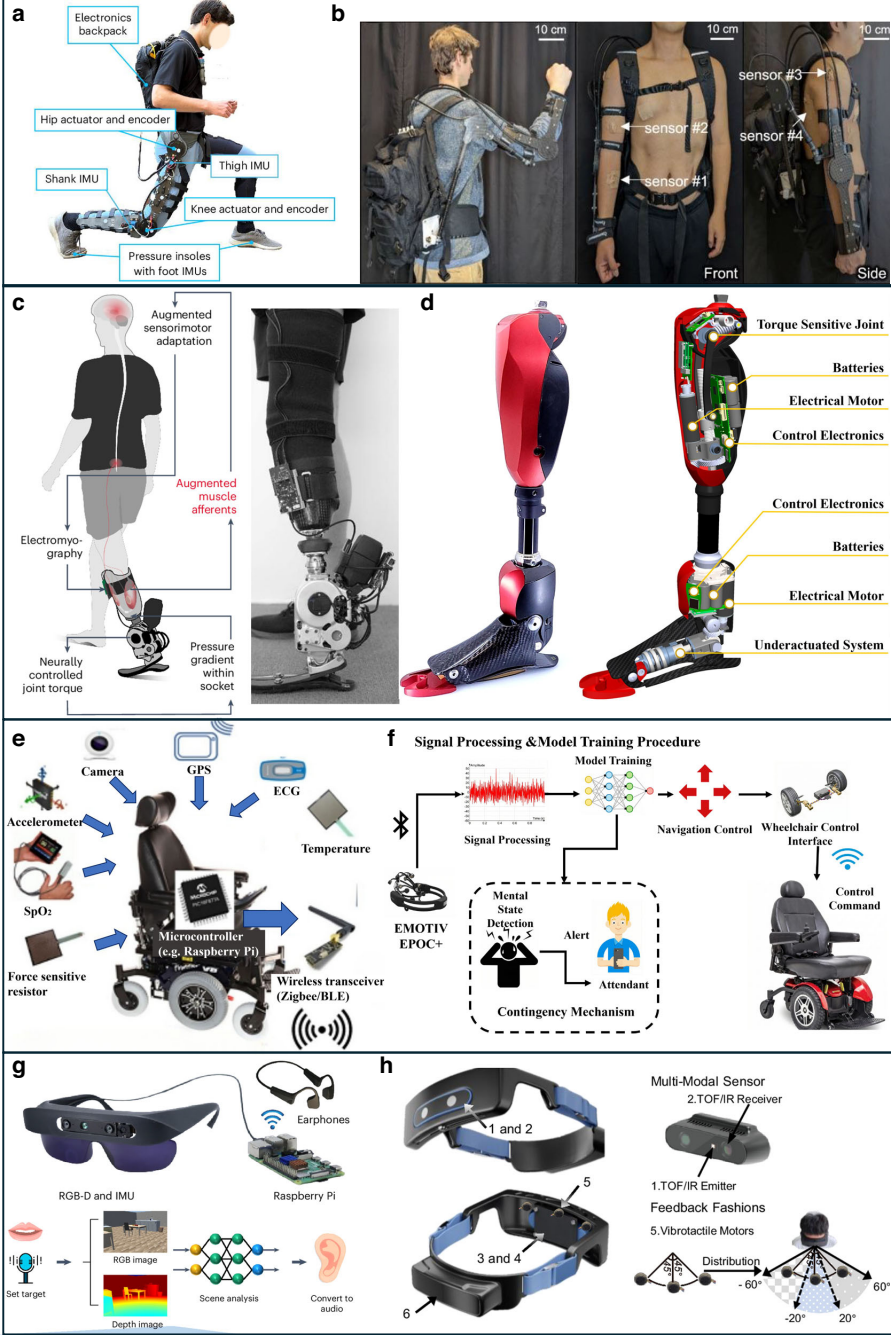
Representative implementations of wearable technologies enabling assisted mobility across different modalities. **a** Exoskeleton using joint moment estimation for task-agnostic support^89^, **b** Upper-limb exoskeleton with soft bioelectronics^45^*;* **c** EMG-driven leg prosthesis for biomimetic gait restoration^107^, **d** A lightweight robotic leg prosthesis replicating the biomechanics of the knee, ankle, and toe joint^108^*;* **e** Multimodal sensing-based smart wheelchair for health monitoring^119^, **f** BCI-controlled wheelchair with adaptive mental-state navigation^118^*;* **g** Visual aid translating depth images to audio for navigation^167^. Reused with permission from Tang et al.^166^*;* **h** Wearable obstacle avoidance system with multimodal feedback^138^.

One of the primary applications of exoskeletons is neurorehabilitation for motor recovery, particularly in conditions like stroke, SCI and multiple sclerosis^84^, which are often present with paresis, spasticity, muscle fatigue, and reduced coordination^84,85^. Exoskeletons enhance motor recovery by enabling intensive, task-specific training that leverages neuroplasticity for motor learning. Commercial lower-limb exoskeletons such as Lokomat, EksoGT, HAL, and Indego support gait training by guiding patients through controlled gait trajectories via actuated hip, knee, or ankle joints^86–88^. These devices incorporate sensors like IMUs and force-sensitive resistors to continuously monitor the patient’s movement intentions and physical condition, allowing real-time, adaptive assistance^89,90^ (Fig. 4a). For example, when a patient initiates a step but lacks sufficient strength, the device provides only the needed torque, promoting active engagement while avoiding over-reliance^91^. This iterative training improves coordination, joint stability, gait symmetry, and walking endurance while reducing compensatory patterns and accelerating motor recovery^87,92,93^. Similarly, upper-limb exoskeletons like ArmeoSpring, MyoPro, and ANYexo 2.0 support rehabilitation of arm and hand movements using adaptive assistance based on muscle activity and biosignals (EEG, EMG), encouraging user participation and functional gains^94–97^.

Despite their potential in rehabilitation, exoskeletons face several challenges. Their rigid structures are often mismatched with complex human biomechanics, resulting in discomfort and unnatural movement^98,99^. Current control systems typically rely on limited inputs such as sEMG or motion capture, hindering accurate, real-time interpretation of user intent^100^. Other significant limitations include high costs, short battery life, bulky design, and complex maintenance^101^. Clinical adoption is further constrained by individual differences in disease stage, gender, height, body size, and muscle tone, affecting both usability and therapeutic outcomes^102^. Future advancement may involve developing soft exoskeletons using flexible biomaterials for enhanced comfort and adaptability^103^. Advances in artificial intelligence (AI) and personalized control algorithms could improve human–machine coordination^104^, while miniaturized actuators may enhance portability and extend battery life^91^. Interdisciplinary collaboration across rehabilitation, neuroscience, and interface design will also be essential for optimizing clinical usability. As regulatory processes evolve and costs decrease, exoskeletons have the potential to facilitate more personalized mobility support.

### Prosthetics for sensory-motor restoration

Prosthetics replace missing limbs entirely, assuming both locomotor and sensory roles^105^, which usually leverages wearable sensing technologies such as EMG, IMUs, and environmental sensors to enable closed-loop, real-time control based on user intent and environmental context^106–108^ (Fig. 4c,d). IMUs and encoders embedded in prosthetic joints enable real-time gait phase detection and adaptive control, improving symmetry and reducing energy cost. Integrated EMG sensors decode user intent for voluntary control of joint movement, supporting both repetitive and complex actions. Environmental sensors such as cameras and LiDAR enhance terrain recognition and obstacle avoidance, improving robustness in real-world use. Integrating soft sensors and biomechatronic components further improves responsiveness, comfort, and long-term usability, making prosthetics a promising direction for next-generation mobility assistance.

Despite these advances, prosthetics still face challenges in achieving stable, high-resolution sensory feedback, particularly during dynamic, real-world tasks^109,110^. Current limitations include signal processing delays, inconsistent sensor performance over prolonged use (e.g., IMU drift), and the need for frequent user-specific calibration. Non-invasive techniques are often limited by low spatial specificity, while invasive methods face biocompatibility, surgical risk, and long-term reliability concerns. Future directions include the development of soft, lightweight sensors to enhance comfort, machine learning models that generalize across users to reduce training burden, and high-efficiency algorithms to improve real-time responsiveness. Co-designing wearable hardware with multimodal sensor fusion can enable robust, personalized, closed-loop control in daily-life environments.

### Smart wheelchair: multimodal biosignal-driven autonomous navigation

Smart wheelchairs expand upon traditional powered wheelchairs to better support individuals with severe motor impairments from conditions such as SCI, progressive neuromuscular disorders, or cognitive disabilities. Unlike conventional wheelchairs, which rely on manual control, smart wheelchairs interpret user intent via physiological signals, reducing effort and improving independence^111^.

Users control smart wheelchairs through various modalities, including EMG, IMU, EOG, and EEG. Wearable EMG sensors placed on accessible muscles translate these signals into directional commands with minimal effort^44^. For example, Oonishi et al.^112^. utilized sEMG signals from the wrist and hand dorsum to control forward and backward motion via a threshold-based disturbance observer. Similarly, IMUs can capture gestures or head movements, enabling intuitive navigation. Mogahed et al.^113^. developed a system for users with quadriplegia that converted head movements into control signals with over 97% accuracy and sub-second response time.

For individuals with severely limited motor function, eye-tracking and BCIs offer non-manual navigation. EOG-based systems detect eye movements and convert them into wheelchair commands, enabling intuitive control. For example, Barea et al. used EOG electrodes to detect horizontal and vertical eye movements, translating them into directional inputs with 95% accuracy^41^. EEG-based BCIs are also commonly used in smart wheelchair control, enabling users to navigate through mental tasks such as MI^114,115^ or SSVEP^116,117^. These methods significantly expand mobility options for individuals with severe motor impairments, highlighting the growing potential of EEG-based smart wheelchairs to foster greater independence and mobility.

Beyond simplifying control, smart wheelchairs integrate real-time physiological monitoring to assess user health and adapt assistance levels accordingly^118^ (Fig. 4f). Embedded sensors track vital signs such as heart rate, muscle fatigue, and respiratory rate^119,120^. ECG and PPG^121^ sensors track cardiovascular activity, detecting issues such as arrhythmia or sudden hypotension. sEMG sensors^122^ assess muscle fatigue, guiding control adjustment to prevent overexertion. Additionally, accelerometers and gyroscopes support fall and seizure detection by recognizing abrupt, abnormal movements and triggering automatic alerts^123–126^. Within shared control frameworks, both the user and system share control authority to allow the user to achieve their goals safely in the environment^127^. When signs of fatigue or reduced engagement are detected from the sensors, the system can temporarily increase autonomy to lessen user effort. This dynamic adjustment minimizes physical and cognitive strain, supporting safer and more sustainable use^128^. With machine learning integration, these systems are also advancing toward predictive analytics, enabling early detection of health risks and proactive assistance.

Despite encouraging progress, smart wheelchairs still face challenges related to user comfort during extended use, power efficiency, signal reliability, and individualized system calibration^119,129,130^. Overcoming these limitations is essential for broader adoptions.

### Non-visual navigation: multisensory fusion for real-time environmental interaction

With ~285 million people worldwide living with vision impairment, including 39 million experiencing moderate to severe blindness^131^, independent navigation remains a significant challenge. Suitable wearable technologies are needed to deliver real-time spatial awareness and enhance environmental interaction. Modern wearable navigation systems integrate real-time sensing, AI-driven data processing, and intuitive feedback to detect, interpret, and communicate spatial information—improving user safety and enabling more autonomous mobility^132^ (Fig. 4g,h).

Effective wearable navigation systems typically involve three key processes: environmental perception, real-time data interpretation, and non-visual user feedback. Environmental perception relies on sensor such as red, green, blue-depth cameras^133–137^, time-of-flight sensors^138,139^, acoustic sensors^140,141^, IMUs^142–144^, electromagnetic sensors^145^ and light sensors^146^ to detect objects, movement and navigation cues like crosswalks and path boundaries^147^ (as shown in Fig. 4g,h). Real-time interpretation employs algorithms, often based on AI to rapidly analyze sensor data and determine precise user location, obstacle distances, and optimal navigation routes^148–150^. Non-visual feedback methods such as auditory signals or haptic (vibrational or tactile) guide users safely through environments without visual cues^141,151,152^. Many systems combine both feedback modalities, enabling adaptability across diverse environments^153^. For example, using haptic cues in noisy urban settings where auditory signals may be less effective^154^.

Wearable navigation devices have been validated in real-world applications with studies confirming their ability to reduce navigation errors and boost user confidence^133,135–137,139,143–145,147,155–162^. Several commercially available systems are already in use: Sunu Band^163^ and BuzzClip^164^ employ sonar-based proximity detection, whereas OrCam MyEye^165^ uses AI and CV to recognize objects, text, and faces, delivering spoken descriptions of the surrounding environment.

Despite technological advancements, current wearable navigation systems still face limitations such as high cost, restricted performance in adverse environmental conditions (e.g., darkness, fog, rain), limited battery life, and a steep learning curve^132^. Additionally, most devices require precise calibration and consistent connectivity, potentially complicating their use^151^. Addressing these issues will require ongoing progress in AI^137^, sensor miniaturization^151^, and user-centered design^166,167^. Enhancing real-time scene comprehension and developing predictive navigation models can improve responsiveness by anticipating movement and suggesting timely adjustments. Moreover, adaptive feedback tailored to user preferences and environmental context could significantly improve usability. With these improvements, wearable navigation systems hold the potential to deliver more autonomous and precise mobility solutions, bridging critical gaps left by conventional aids for the visually impaired.

## Outlook

### Current landscape and unmet needs

Ageing-related mobility decline constitutes a societal-scale challenge that complements disease- and injury-related rehabilitation needs. According to the World Population Prospects 2024 Revision, the global population aged 65 and overreached ~809 million in 2023^168^. Projections further indicate that by 2035 China alone will have over 300 million citizens above 65 years, accounting for more than 20% of its population^169^. These demographic shifts underscore the urgency of addressing ageing alongside clinical rehabilitation. Clinical disorders represent urgent scenarios requiring therapeutic devices, while ageing reflects a broader demographic demand for long-term mobility support. Regulatory frameworks further highlight this distinction: post-stroke exoskeletons are typically subject to stringent medical device approval, whereas fall-prevention sensors or balance-support exoskeletons for older adults may be classified as wellness or assistive devices with fewer regulatory barriers. Importantly, technologies developed for rehabilitation can serve as a foundation for ageing-related applications. For example, a wearable hip exoskeleton validated in post-stroke gait training for improvements in gait parameters and muscle effort has subsequently been shown to support daily physical activity and gait exercise in older adults. Such shared sensing and control modules can be leveraged to reduce development costs and facilitate broader adoption across populations^170,171^.

The global burden of mobility-related impairments remains substantial and unevenly distributed. Current solutions, such as robotic exoskeletons, prosthetics, smart wheelchairs and non-visual navigation, have shown significant promise in restoring mobility and autonomy across diverse settings, including rehabilitation clinics and home-based care. However, many of these systems face persistent technical limitations that hinder widespread adoption. Sensor data can be susceptible to distortion and noise^10^, while limited battery life^10,172^ makes it challenging to maintain a continuous power supply for long-term monitoring. A further challenge lies in the inequalities in sensing accuracy and accessibility across populations, particularly in modalities such as EEG^173^, PPG^174^, and ECG^175^. Participatory co-design is also critical for the successful development and adoption of assistive mobility technologies^176^. By involving users directly, it ensures solutions are usable, sustainable, and aligned with real-world needs^176,177^. For example, Biggs et al. demonstrated how participatory workshops with blind and low-vision travelers refined non-visual navigation cues to better fit everyday wayfinding practices, underscoring the value of user involvement in shaping technical features^178^. The SOC framework (Selection–Optimization–Compensation) complements this approach, highlighting that technologies should not only compensate for functional loss but also optimize residual abilities. In exoskeleton research, human-in-the-loop optimization strategies exemplify this principle by iteratively tuning assistance profiles to individual gait dynamics, thereby enhancing both efficiency and safety^179^. Similarly, navigation systems can be designed to guide users along safer or more accessible routes^180–182^. Combining co-design with SOC principles has the potential to strengthen user acceptance, destigmatize device use, and expand the role of these systems in supporting both recovery and proactive adaptation.

Contextual metadata, such as environmental conditions, spatial information, and user preferences, can enhance wearable mobility systems by enabling adaptive navigation, informed behavioral adjustments, and improved safety and usability^183^. For instance, an outdoor navigation system for blind users integrates GPS with cartographic data to deliver spatialized audio cues, demonstrating how environmental and spatial metadata can be translated into real-time, user-friendly guidance^184^. Beyond environmental data, behavioral and psychological information such as ecological momentary assessment (EMA) can also provide valuable context for system design, supporting the development of more adaptive technologies and facilitating participatory co-design with users^185,186^.

Beyond the technologies discussed above, other commonly used assistive devices, such as rollators, have not yet been extensively studied in conjunction with wearable sensors^187,188^. Future advancements that integrate these assistive devices with wearable sensors could enable better monitoring of users’ movement patterns, enhance risk detection, and provide more personalized support, thereby improving their overall effectiveness as mobility aids^188^.

Across the adoptions of wearable technologies, each technology also faces specific issue. Exoskeletons are constrained not only by delays between intention recognition and actuation but also by weight, bulk, and limited adaptability across diverse daily activities^88^. Prosthetics suffer from delays in integrating user intention and sensory feedback as well as challenges in achieving naturalistic multi-degree-of-freedom control^189^. Non-visual navigation systems require intuitive, low-burden feedback in complex environments and remain sensitive to environmental variability such as lighting or weather^166^. Smart wheelchairs are restricted by limited command sets, high cognitive demands, and difficulties in operating within dynamic, cluttered environments.

Shared challenges include high power consumption, susceptibility to sensor noise, privacy concerns associated with sensitive data, and the need for extensive user customization and training, which together hinder broader adoption^88,166,189^. Addressing these issues requires both cross-cutting and device-specific strategies. For example, deep learning and computer vision methods can improve environmental perception for non-visual navigation and smart wheelchairs, while enhancing intention recognition for exoskeletons and prosthetics, and simultaneously filtering sensor noise^45,190,191^. Advances in low-power electronic components such as memristors can substantially reduce energy consumption, and edge processing architectures can protect user privacy by enabling on-device computation without reliance on remote servers^192–194^. Finally, embedding co-creation design principles throughout development can address user customization and training needs, ensuring that systems are not only technically robust but also tailored to everyday practices and user acceptance, thereby optimizing overall product experience^176,177^. The advancements of wearables for assisted mobility reflect a paradigm shift, from isolated, function-specific devices to intelligent, connected, and user-centric systems.

Additional barriers for real-world use include high costs, short battery life, discomfort, and a lack of widely available commercial products. Addressing issues of usability, affordability, and continuous operation is essential to support broader adoptions, especially in low-resource settings. Emerging technologies are expanding what wearable systems can do. Besides this, although the potential of wearable devices for mobility enhancement is widely recognized, there is a lack of a unified framework for evaluating their effectiveness, usability, and long-term impact. For exoskeletons, prostheses, smart wheelchairs, and non-visual navigation systems, each subfield relies on individual and task-specific evaluation methods. There is currently no universally acknowledged standard for assessing system performance. The difficulty of establishing a unified evaluation framework lies in the high heterogeneity of user populations and needs. Widely used scales, such as the Jebsen–Taylor Hand Function Test and the Box & Block tests, provide objective scores on specific functional dimensions and normative data for clinical or research contexts, but they fall short of capturing requirements across diverse devices and user groups^195^. For instance, in upper-limb prosthetic rehabilitation, Resnik et al. found that outcome measures such as the Jebsen–Taylor and Box & Block tests show variable responsiveness across different levels of amputation^195^. Moreover, assistive devices are typically highly personalized with numerous adjustable parameters. Even for a single prosthetic device, the interpretation of performance metrics (e.g., when using multiple measures of control, gait, user experience with varying weightings) continues to evolve, highlighting the immaturity of internal evaluation frameworks and the challenge of cross-industry harmonization^196^. Data collection and sharing further face ethical and privacy barriers. Wearable and digital health research repeatedly report users’ reluctance to share sensitive physiological and behavioral data, inconsistencies in policy implementation, and the lack of unified inter-institutional data-sharing agreements^197^. These constraints directly limit the availability of open datasets and cross-context benchmarks, thereby restricting the development of a standardized framework. Establishing standardized evaluation frameworks thus represents an important direction for future work and requires joint input from researchers, clinicians, and end-users throughout the design and implementation process. Critical questions, such as how to optimize devices for specific types of mobility impairments, how to balance performance with user comfort, and how to ensure the affordability and accessibility of these technologies, remain underexplored and not addressed.

Ongoing interdisciplinary research and continued technical improvements will be essential to overcome these barriers. By fostering collaboration across engineering, medicine, and user-cantered design, the next generation of wearable assistive technologies are expected to move beyond technical innovation toward greater autonomy, safety, and quality of life for users worldwide.

### Human in the loop: challenges in sensorimotor-cognitive integration

Despite the sophistication of embedded sensors or control algorithms, the human brain remains a vital limiting factor. Whether the goal is to augment motor capacity (as in exoskeletons), restore (prosthetics), or replace lost function (as in wheelchairs), effective real-world performance depends on the user’s ability to integrate these devices into their existing sensorimotor and cognitive systems to manage novel input–output mappings and adapt to unfamiliar sensorimotor contingencies. Technologies that appear intuitive on paper may collapse under real-world conditions where users must simultaneously coordinate multiple goals, as seen with midair haptic systems, which often produce faint signals that require concentrated attention and thus underperform in complex everyday contexts^198^.

This integration is non-trivial. As demonstrated in upper-limb augmentation research using extra robotic digits and limbs, introducing artificial actuators—even in able-bodied users—requires the brain to recruit control strategies and sensory mappings that are not innately available^199^. In such cases, users must “borrow” neurocognitive, sensory and motor resources from other body parts (e.g., toes controlling a robotic thumb^200^), leading to what has been termed the resource allocation problem: the cognitive and neural cost of operating a new device without compromising existing function^199^. For example, controlling a robotic thumb using the toes may impact fundamental lower limb function, as recent evidence shows that both actively using and merely wearing the toe-controlled robotic thumb can lead to measurable declines in balance performance, suggesting competition with the toes’ original role in postural stability^201^.

Crucially, this challenge is not unique to augmentation^202^. It generalizes to assistive mobility technologies where the user must continuously plan, monitor, and adapt their interaction with the device under conditions of physical impairment, environmental uncertainty, and cognitive load. The human sensorimotor system evolved efficient strategies—like sensory gating, attenuation^203^ and active inference^204^ —to reduce redundant input and prioritize error signals^205^. Artificial feedback systems often bypass these mechanisms, delivering non-adaptive, uniformly salient signals. Even systems designed for simplicity, like vibrotactile or visual feedback cues, may become counter-effective if they are not congruent with the brain’s filtering and predictive mechanisms. For example, continuous vibrotactile cues can induce sensory overload or desensitization, leading users to ignore the feedback altogether^198^.

Paradoxically, the challenge of neurocognitive compatibility for motor interfaces might grow with the sophistication of the wearable interface, such as when artificial systems generate high-dimensional or ambiguous data streams that must be interpreted in real time. Even interfaces designed to be highly intuitive may falter if they approximate—but fail to fully replicate—natural sensorimotor mappings^206^. For example, near-biomimetic sensorimotor interfaces can produce mismatches between expected and actual sensations, triggering challenges analogous to the “uncanny valley” in artificial vision^207^.

In addition, temporal delays—whether introduced during sensing, processing, or actuation—pose a critical barrier to seamless integration. The human sensorimotor system relies on tight timing loops, often on the order of tens of milliseconds, to predict and correct movement. For example, temporal delays as short as 50 ms in haptic cues have been shown to distort perceived stiffness and object cohesion^208^. Beyond disruptions for motor control, delays exceeding this threshold can disrupt the user’s sense of agency, making actions feel disconnected from intention^209^. This issue is especially acute in interfaces that depend on slow signal acquisition (or accumulation) and processing, such as EEG or fNIRS, where effective control signals may take up to hundreds of milliseconds to emerge. These lags impair the fluidity of control and can undermine the user’s confidence in, and sense of ownership over, the device’s actions.

Finally, we must acknowledge that neurocognitive integration is context-dependent^198^. Physiological and cognitive factors like fatigue, stress, cognitive load and divided attention can significantly alter how an individual will act and react. A haptic cue that is helpful during training in a lab may become irrelevant or even misleading when navigating a crowded, noisy urban environment. The cognitive effort required to control a neural signal for EEG and fNIRS interfaces might be too costly when trying to multitask in everyday settings. Indeed, mobile fNIRS studies show that under dual-task conditions, performance declined sharply and prefrontal activation plateaued or even dropped^210^. Thus, rather than focusing solely on biomimicry or technical fidelity, future co-creation designs must prioritize technologies that adapt to human cognitive variability and offer flexible, context-sensitive modes of interaction.

## Conclusion

This perspective provides a timely report of the status and prospects of the rapidly evolving area of wearable technologies in assisted mobility, informing a guidance for its future development. In the context of global population ageing and global economic slowdown cycles, there is a clear need that the wearable mobility technologies in the real world should be more accessible, inexpensive, inclusive, intelligent, user-centric, and personalized, and be able to effectively “close the human loop”. Nonetheless, given the rapid progress in wearable technologies, co-creation design, and medical engineering, there is strong reason to believe that meaningful improvements in assisted mobility—and, consequently, in the independence and quality of life of individuals with severe motor impairments—can be realistically achieved in real-world settings within the next decade.
